# The first case of *Brucella canis *in Sweden: background, case report and recommendations from a northern European perspective

**DOI:** 10.1186/1751-0147-54-18

**Published:** 2012-03-27

**Authors:** Bodil Ström Holst, Karin Löfqvist, Linda Ernholm, Karin Eld, Maria Cedersmyg, Gunilla Hallgren

**Affiliations:** 1 Department of Animal Health and Antimicrobial Strategies, National Veterinary Institute, SE-751 89 Uppsala, Sweden; 2 Department of Clinical Sciences, Swedish University of Agricultural Sciences, Box 7054, SE-750 07 Uppsala, Sweden; 3 Helsingborg Animal Hospital, Box 220 97, SE-250 23 Helsingborg, Sweden; 4 Department of Bacteriology, National Veterinary Institute, SE- 751 89 Uppsala, Sweden; 5 Swedish Board of Agriculture, 551 82 Jönköping, Sweden; 6 Department of Disease Control and Epidemiology, National Veterinary Institute, SE-751 89 Uppsala, Sweden

**Keywords:** Brucella canis, Canine abortion, Reproduction, Canine brucellosis

## Abstract

Infection with *Brucella canis *has been diagnosed in Sweden for the first time. It was diagnosed in a three-year-old breeding bitch with reproductive disturbances. Fifteen in-contact dogs were tested repeatedly and all of them were negative for *B. canis*. The source of infection could not be defined. The present article describes the case and the measures undertaken and gives a short review over *B. canis*. Recommendations on how to avoid the infection in non-endemic countries are given.

## Background

Until 1994, Sweden had a quarantine requirement for all dogs entering from abroad, except for dogs from Norway. Norwegian dogs were allowed to enter without any requirements at all since Norway has had the same epidemiological status as Sweden for several decades. From 1994 to 2004, however, dogs from EU and EFTA countries were allowed entry without quarantine if in possession of an identity mark, rabies vaccination, rabies antibody titre control and certain other prophylactic treatments. An import license, amongst other documentation, was required. In 2004, the new European pet regulation 998/2003 came into effect allowing entry in combination with certain national requirements. For dogs from EU and EFTA countries only identity marking, rabies vaccination, rabies antibody testing, deworming and a pet passport was needed. These changes in regulations facilitated travelling of dogs between Sweden and other European countries, especially those within the European Union (EU). In addition, it led to an increased responsibility for dog owners to protect their travelling dogs against unfamiliar infections that did not exist in Sweden.

**Figure 1:**
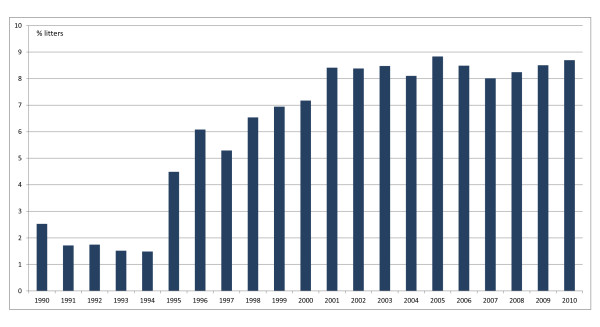
**The proportion of litters registered from stud dogs not registered in Sweden or Norway (per cent of total number of litters registered in Sweden)**.

Dogs travel with their owners for varying reasons, such as holiday, dog shows or breeding. The number of litters that are registered within the Swedish Kennel Club from a stud dog that is not registered in Sweden or Norway has increased since 1994, and is today around 8% of the total number of litters born, or approximately 1000 litters per year (Figure 1). Breeding leads to a special risk for transmission of infectious diseases due to the close contact between the pair during mating. *Brucella canis *is of special concern for breeding dogs, since it can be transmitted venereally and cause reproductive problems. There are reports that dogs can also be infected with other *Brucella *species: *Brucella melitensis *[1], *Brucella suis *[2] and *Brucella abortus *[3], but it is only *B. canis *that is of epidemiological importance to the canine population.

### Prevalence of *B. canis*

**Figure 2:**
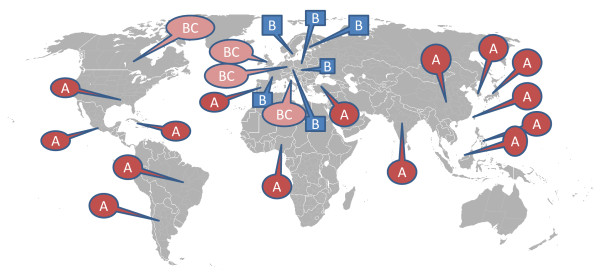
**Geographical distribution of *B. canis*, clinical cases and seropositive dogs**. A: Reports (including serosurveys) indicate that the infection is endemic. B: Few clinical cases or outbreaks in kennels have been described C: Serosurveys indicate that the seroprevalence is low.

Infection with *B. canis *is common in Central and South America and in southern USA [2,4] and has also been reported from Canada [5,6]. In the USA, the dog trade has led to a spread of the infection, and a growing need for regulatory tools has been addressed [7]. In Georgia, infection of dogs with *B. canis *was notable in 2003, and a control strategy for the infection developed [8]. In Asia the infection has been described and studied in Japan [9,10], and it has also been reported from India [11], the Philippines [12], Korea [13], China [14], Malaysia [15] and Taiwan [16], and in Africa from Nigeria [17]. The number of described cases from central Europe is low, but cases may be unrecorded since *B. canis *is neither notifiable to the World Organisation for Animal Health (OIE) nor to the EU. Stray dogs in the Mediterranean area are suggested to serve as a reservoir [18,19]. In recent years, the number of published reports on *B. canis *infection has increased. In Finland the infection has been diagnosed from dogs that were imported from Russia [20]. The first outbreak in Hungary of *B. canis *in a kennel was recently described [21], and cases in male dogs are reported from Italy and Germany [22,23]. Infection with *B. canis *was recently described in three poodle bitches in a kennel in Austria [24]. Earlier cases in Austria were a mongrel dog from Greece and a purebred male dog [24]. Antibodies to both *B. abortus *and *B. canis *have been described in a male dog in Poland [3]. There has been serological evidence that *B. canis *is present in Great Britain [25], Germany [26-28], Italy [29] and Spain [19]. One case from Great Britain is reported in a dog that was imported from Spain [30]. In a dog shelter in Turkey, 4 out of 48 dogs (8%) that had died of unknown reason were found to be infected with B. *canis *[18]. In Europe, infection with *B. canis *has also been reported in laboratory dogs in former Czechoslovakia [31] and from a kennel in France [32]. A schematic drawing of prevalence of *B. canis *is shown in Figure 2.

### Pathogenesis

*B. canis *enters the body via the mucosal membranes of the oropharynx, genital tract or conjunctiva [33,34]. The bacteria are then phagocytised by macrophages and other phagocytic cells, and transported via the blood to lymph nodes, spleen and genital organs, where they multiply [33]. Hyperplasia of the lymphoid tissue is seen throughout the body [35]. Bacteremia occurs 1 to 4 weeks after the infection and persists for at least 6 months, and then reoccurs intermittently for up to 5 years [36].

### Clinical signs

The most typical clinical sign is late-pregnancy abortion, week 7-9, [37], and this was the first sign described to be related to infection with *Brucella canis *when the bacterium was first recognized, in 1966 [38]. Aborted pups are partially autolysed and show signs of a generalised bacterial infection, such as subcutaneous oedema, abdominal hemorrhages, and degenerative lesions in liver, kidneys, spleen and intestines. The bitch continues to excrete a brownish or green-gray discharge for 1-6 weeks after the abortion [37]. Sometimes early embryonic death and resorption occurs. Weak pups may die within a few hours, but other times may survive up to a month. Seemingly normal pups can also be born, and develop the disease later [33]. Before puberty, a generalised lymphadenopathy is the most common clinical sign. Brucellosis does not affect the oestrous cycle, and bitches that have aborted can give birth to normal litters in a subsequent pregnancy, or have intermittent reproductive disturbances [33].

In male dogs, epididymitis and prostatitis are the most common clinical signs. In the acute stage, the epididymis increases in size and is painful. Frequent licking may lead to scrotal oedema and dermatitis. In the chronic phase the epididymides may become small and hard, and the testicle atrophic. Orchitis and ulcerative scrotum dermatitis can be seen [39]. Chronically infected dogs are often oligo- or azoospermic, and infertile. Sperm defects that can be seen include tail defects, loose heads and distal droplets [40]. In the testicles, tubuli are fibrosed [41]. The testicular damage leads to production of auto-antibodies towards the sperm cells. These antibodies can be detected in serum and seminal plasma from three months after the infection [42,43]. From 4 months after infection the sperm cells autoagglutinate [42].

Most often there are no general clinical signs, and infected dogs do not usually have a fever. Sometimes the dogs have a dull coat, or show decreased exercise tolerance, but this is not common [44]. Lymphadenopathy of the retropharyngeal or inguinal lymph nodes, or generalised lymphadenopathy sometimes occurs [33]. Discospondylitis has been reported [45,46]. Dogs that were experimentally infected with *B. canis *often showed recurrent uveitis for several months after the infection [40] and there are several case reports describing ocular signs, such as endophthalmitis and anterior uveitis, in dogs infected with *B. canis *[47-49]. Osteomyelitis related to hip prostheses has been described in two dogs [50]. Chronic meningitis and non-suppurative encephalitis has been associated with bacteria belonging to the genus *Brucella *[33].

### Diagnosis

The diagnosis is made by culture, polymerase chain reaction (PCR) or serology, often in combination. To make a definitive diagnosis by bacterial culture, the dog should not be treated with antimicrobials. Blood culture can be used, but often genital samples are better, especially if the dog shows clinical signs of the genital tract. *B. canis *may be cultured from semen and vaginal discharges during oestrus or after abortion and a high concentration of the infecting agent can be found in the placenta. Urine cultures can be positive even if a blood culture is negative. The highest urine concentration is seen 8-12 weeks after infection, with the concentration in urine being higher from male dogs than from bitches [51]. The organs most suitable for culture, from biopsies or at post mortem examination, are lymph nodes, prostate and spleen [26]. PCR-analysis of whole blood has a high sensitivity, whereas serum is not suitable for PCR analysis [52]. Semen can be analysed with PCR [53,54]. PCR can be a more sensitive method than bacterial culture because it detects not only viable but also dead bacteria, and because the method is not affected by contamination with other bacteria [55].

For screening, serologic tests are used. A rapid slide agglutination test, RSAT, is commonly used, although a drawback to this method is the risk of false positive samples. By treating the sample with 2-mercapto-ethanol (2-ME) the number of false positive samples decreases [19,56] because IgM is dissociated and IgM cross-reacts with other bacteria more commonly than IgG [57]. The agar gel immunodiffusion (AGID) test has been described as suitable if a chronic infection is suspected, because more chronic infections are positive using an AGID than using other tests [36]. However, the AGID is not used by many laboratories today because of its low sensitivity and because it requires trained personnel and special media [8]. Enzyme-linked immunosorbent assays (ELISA) have been developed [58] and a lateral flow immune-chromatographic assay (LFIA) has recently been described [59]. A combination of different serological tests is often recommended.

### Treatment

Different antibiotics have been effective against *B. canis *in *in vitro *studies [1,60], but multiresistant strains have also been described [61]. Treatments that have been evaluated include 1-3 weeks of combinations of different antibiotics such as streptomycin, tetracycline, rifampicin and sulphonamides [62,63], two weeks' treatment each with tetracycline, followed by dihydrostreptomycin and trimetoprim-sulfadiazine [64], or 30 days of treatment with enrofloxacin [65]. Clinically, no antibiotic treatment has been shown to eliminate the infection in all treated animals. If *B. canis *is diagnosed in a kennel, the affected animal should be isolated and not used for breeding. The environment should be thoroughly cleaned and disinfected. Since an infected dog, even if it is clinically healthy, can infect other dogs and humans, euthanasia is often recommended.

### Transmission

Dogs are most commonly infected by contact with vaginal discharges at oestrous or after abortions, or by ingesting infected placentas or foetuses. At abortion, the placenta and the discharges can contain up to 10^10 ^colony forming units (cfu) per mL, and the oral infection dose is 2 × 10^6 ^cfu. Experimentally, 10^4 ^cfu has been sufficient for conjunctival infection [34,40,66]. Thus, 1 mL placental tissue or vaginal discharge is equal to approximately 100 000 infectious doses, and the bitches can have a vaginal discharge for up to 6 weeks after an abortion. Dogs can also be infected at mating. It should be observed that chronically infected dogs can be serologically negative and negative on blood culture, although *B. canis *can be detected from the prostate, epididymis and semen, or in female dogs in vaginal secretion, and thus they can still infect other dogs at mating or via artificial insemination [36,54,67].

Environmental infection is possible, especially from areas where dogs often urinate [34], or where vaginal discharges are deposited. Dogs living together are at risk of infecting each other. Within half a year, infected male dogs had infected other male dogs, and bitches had infected other bitches [51]. It was suspected that contaminated urine was an important source of infection in these cases, especially from male dogs. The concentration of bacteria in urine and semen is highest from 1 to 4-6 months after infection [51].

### Recommendations to avoid introduction of *B. canis *into non-endemic areas

To avoid introducing brucellosis into a kennel, the introduction of untested dogs from endemic areas should be avoided. Dogs from kennels in which *B. canis *has been diagnosed should not be used for breeding (Table 1). Dogs from endemic areas should be kept isolated until tested free of *B. canis *to avoid further spread of the disease. This is recommended for natural mating, artificial insemination with fresh, chilled or frozen semen, and when introducing new dogs into the kennels. For kennels in endemic areas it is often recommended that breeding animals are tested annually, and that all new dogs are tested before being introduced into the kennel. Serologic tests can be negative up to 4 weeks after infection, and at least 12 weeks must pass to be sure of detecting antibodies in an infected animal [36]. Therefore, two negative tests 4-6 weeks apart are needed in case the dog is incubating the disease [8]. At least one of the sampling occasions should be no earlier than 12 weeks after suspected contact with an infected animal. Bitches that might be chronically infected should be tested during oestrus, pregnancy or at abortion, as they may have low antibody titres at other times [44]. Chronically infected male dogs can also be difficult to detect, as they too can be serologically negative. In addition to serologic methods, bacterial culture or PCR analysis can be performed to detect the bacterium. Only dogs tested free from *B. canis *should be used for breeding, and recommended tests depend on the level of risk (Table 1).

**Table 1:** Recommendations for testing dogs from areas endemic for *B. canis *when mated or inseminated with dogs from non-endemic areas, or when imported to non-endemic areas

Risk level	Situation	Recommended test
1	Mating or insemination, no suspicion of *B. canis *and no reproductive disturbances	Blood sample for antibody analysis

2	Mating or insemination with dog with previous reproductive disturbances, import of dogs without reproductive disturbances.	Serology: 2 samples 4-6 weeks apart

3	Import of or mating/insemination with dogs with previous reproductive disturbances, infection with *B. canis *suspected.	Serology, 2 samples 4-6 weeks apart, blood culture and culture or PCR from semen or vagina

4	Import of or mating/insemination with dog from kennel with endemic infection of *B. canis*.	Dissuaded from mating or import.

If *B. canis *is detected in a group of dogs, regular testing and removal of infected dogs and strict hygiene is necessary to eradicate the infection [28]. Common disinfectants are effective against *B. canis*.

### Infection with *B. canis *in humans

Humans can become infected with *B. canis*, although reported cases are scarce. The clinical presentation is unspecific, including fever (as opposed to the disease in dogs), headache and fatigue, and a history of exposure to dogs is usually needed to raise suspicion of infection with *B. canis *[68]. The infection may be underdiagnosed due to its rather unspecific symptoms. Positive blood cultures confirm the diagnosis, but as the patients have been treated with antibiotics in many cases, the risk of a false negative culture is increased [69]. Furthermore, *B. canis *is slow-growing and mildly fastidious, producing low-grade bacteremias, decreasing cultural sensitivity [68]. Serodiagnosis is problematic due to a lack of antigenic cross-reactivity with the antigens routinely used, and a case that was serologically negative for *B. canis *despite positive blood culture has also been described [70]. Treatment with antibiotics is usually effective in humans.

There are several reports on disease in humans without immune disorders. A 17-month old boy had refusal to walk as the chief symptom [71]. An outbreak with fever, diarrhoea and vomiting in children and headache, fatigue, myalgia and nausea in adults has been described [72]. Fever, fatigue, and nausea are common clinical signs [73,74]. A man with endocarditis was serologically positive for *B. canis *although the source of infection could not be established [75]. A fifteen year old boy suffered from weakness, persistent fever and lymphadenopathy [76]. Sometimes relapses and more prolonged courses of illness are described [69].

Clinical infection with *B. canis *has also been reported in persons with immune disorders. Signs such as fatigue, fever, malaise, headache, cough and arthralgia have been described [77,78].

## Case presentation

In December 2010, a 3-year-old American Staffordshire terrier imported from Poland, expecting her first litter, was brought to the Regional Animal Hospital of Helsingborg, Sweden. She was 46 days pregnant but although her general condition was good, she had a green vaginal discharge. An ultrasonography was performed which revealed that all pups were dead. The bitch was aborted with aglepristone and prostaglandins, and also received amoxicillin. Hematology showed mild leukocytosis and moderate eosinophilia. Bacterial culture from the vagina showed no bacterial growth. The bitch was monitored with ultrasonography and clinical examinations for the following weeks. Her general condition was good and the discharge ceased rapidly. No foetal parts could be identified in the discharge.

In the follow-up of the abortion the owner reported that the bitch had been mated in Poland, and that she had been mated once previously with a dog from Serbia, although she did not conceive at the time. This led to the suspicion of brucellosis, and the bitch was tested serologically for *B. canis *in January 2011, with a negative result. An analysis for Giardia and a Baermanns flotation test for parasites were also negative. The owner was recommended to vaccinate the bitch against canine herpesvirus at the next mating, and to follow progesterone levels during the next pregnancy.

In the end of May 2011 the bitch was again presented at the animal hospital. She had been taken to Poland for mating to the same dog again, and now had a bloody discharge at day 59 of pregnancy. Ultrasonography was performed and all but one pup were dead. A caesarean section was performed, but no pups were alive. Ten dead, partially autolysed pups were delivered. The uterus was filled with a brown-greenish discharge and all pups had cutaneous ulcerations. The bitch was treated with amoxicillin and cabergolin, and recovered uneventfully. This second abortion in the last trimester in a clinically healthy bitch mated abroad again raised the suspicion of brucellosis, and the placentae from aborted foetuses were sent to the National Veterinary Institute (SVA) for bacteriological analysis, specifically asking for *B. canis*.

The culture result from the placentae was positive. As this was the first case of *B. canis *in Sweden, the strain was sent to EU-RL (European Union Reference Labaratory) in Alfort, France, for confirmation. The bitch was also tested serologically, and was now positive for *B. canis*. The Swedish Board of Agriculture (JV) decided that the bitch had to be isolated until a definitive diagnosis was made (confirmation by EU-RL); they initiated contact tracing and contacted the county veterinarian and the county medical officer. The bitch was tested again by blood culture, vaginal culture and serology (RSAT). *B. canis *grew in the blood culture and in the vaginal culture, and the bitch still had antibodies to *B. canis*. The first bacteriological culture was confirmed by the EU-RL. The Swedish Board of Agriculture decided that the bitch should be euthanized, as recommended by SVA. The bitch was euthanized at the Regional Animal Hospital of Helsingborg.

### Contact tracing

SVA recommended how and which dogs to test, and JV had the main responsibility for the contact tracing. Dogs within the country that had been in contact with the infected bitch through mating, common housing or other close contact, were tested. All in-contact dogs were tested twice, with cultures from blood and the genital tract, and by serology. At least one of the occasions should be minimum 12 weeks after the latest risk contact. At least one sample from the genital tract should be during oestrus in bitches, and semen was cultured from the male dogs.

**Figure 3:**
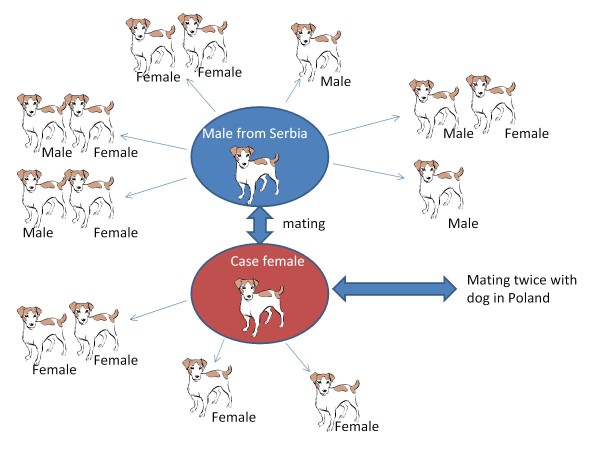
**Schematic figure describing contact tracing**. The bitch within the red circle is the one diagnosed with *B. canis*. Male dog within blue circle: the first dog (from Serbia) that mated the bitch and that now lives in Sweden. Thick arrows: contact through mating. Thin arrows: Dogs that had other close contact. Dogs that lived together in the same household are placed together.

Possible sources of infection included the two dogs that had mated the bitch. The first dog that had mated the bitch, when she did not conceive, had been sold to Sweden by this time and was a stud dog in northern Sweden. There was a history of bitches not conceiving after mating with him. A semen sample in October 2010 revealed azoospermia, macrophages and epithelial cells in the small ejaculate of 1 mL. In July 2011 the total sperm count was 115 million, with 60% sperm having various unspecified defects and an abundance of epithelial cells. Two months later, in September, the total sperm count was 1265 million, 75% progressive motility and 10% unspecified defects. In November 2011 a new semen sample was taken and evaluated. The total number of sperm was good (1700 million), their motility was good and defects were within normal limits. Epithelial cells and leukocytes were also within normal limits. He was tested for *B. canis *in July, September and November 2011 by blood and semen culture and by serology (RSAT), and in November semen was also analysed by PCR. In-contact dogs to this male dog were tested in the same way as in-contact dogs to the bitch. In total, 15 in-contact dogs of different breeds were tested with no test results being positive. The contact tracing is schematically described in Figure 3.

In Poland, the male that mated the bitch the second and third time was serologically tested (RSAT) twice during the autumn 2011, with negative results, and he is still being used for breeding in Poland. He resides in a kennel. The *Brucella *status of the other dogs is not known.

## Discussion

The bitch was imported as a young dog to Sweden from Poland. The first male dog that she was mated to, from Serbia, has a history of bitches not conceiving after mating, including the present bitch. There are several causes for bitches not to conceive, including poor semen quality due to infection with *B. canis*. This male dog has been included in the contact tracing as he now resides in Sweden.

At the second and third mating, a male dog from Poland was used. The bitch conceived after both these matings, but aborted late in pregnancy, which is typical for infection with *B. canis *[44]. Autolysed pups with subcutaneous hemorrhages are typical and could be seen in the present case. The vaginal discharges had a short duration, possibly due to the fact that the bitch was treated with amoxicillin after both abortions.

The bitch was serologically negative for *B. canis *in January 2011, after the first abortion. More than 12 weeks had passed since the bitch mated the first male dog, and 11 weeks since she mated the second male, from Poland. In addition she was sampled close to an abortion, which stimulates antibody production in chronically infected bitches. No bacterial cultures were carried out at this occasion. The suspicions were directed against the male dog from Serbia, because bitches had failed to conceive after mating with him, whereas the other male dog had sired healthy litters. Extensive testing of the imported dog from Serbia has been carried out, and all results were negative for *B. canis*. His semen quality, although previously was poor, has normalised. The second male dog has been tested twice by serology, and also found negative. The source of infection for the bitch could thus not be established.

Brucellosis in food-producing animals in Sweden is regulated by law and the costs for measures against the disease, including compensation to the animal owner, are paid by the government. The reasons for the government to act to eradicate the infection in a dog were a combination of the fact that *B. canis *is a zoonosis and that a quick and reliable diagnosis is missing, thereby making it difficult for owners to protect themselves and their animals, should the infection be established within the country. International experiences show that powerful measures, such as euthanasia of infected animals, is often needed to eradicate the infection in kennels and to stop further spread of the infection [8].

### What are the consequences for the pet owners?

The diagnosis of *B. canis *in the bitch was a personal tragedy to the owner. She lost her dog, money and future pups. She was a small-scale breeder; in larger kennels the economic consequences will be even bigger as all breeding stock may be lost. The consequences were considerable also for the owners of in-contact dogs. They had to keep their dogs separate and bring them to animal clinics or hospitals for sampling, and could not use them for breeding until they were declared free from *B. canis*.

## Conclusions

A typical case of *B. canis *in a dog with reproductive disturbances has been diagnosed in Sweden. Handling of the case by the practicing veterinarian and by the authorities is described. Results from the contact tracing indicated that the infection most probably has not spread within the country. The source of infection could not be identified. Recommendations on how to avoid introduction of *B. canis *into non-endemic areas are given.

## Abbreviations

AGID: Agar gel immunodiffusion; CFU: Colony forming units; ELISA: Enzyme-linked immunosorbent assay; LFIA: Lateral flow immune-chromatographic assay; 2-ME: 2-mercapto-ethanol; PCR: Polymerase chain reaction; RSAT: Rapid slide agglutination test.

## Competing interests

The authors declare that they have no competing interests.

## Authors' contributions

BSH compiled the information, initiated and drafted the manuscript, KL handled the case including communication with authorities and drafted the manuscript, LE and GH handled the contact tracing at the National Veterinary Institute and MC was responsible at the Swedish Board of Agriculture. KE was responsible for the bacteriological parts. All authors read and approved the final manuscript.
